# 

**Published:** 1906-10-06

**Authors:** 


### Two Indispensable Handbooks for Nurses

JUST READY. NEW AND REVISED EDITION.

### SURGICAL INSTRUMENTS AND APPLIANCES.

1/6 net, 1/8 post free.

An Illustrated and Classified List, with Explanatory Notes.

CONTAINING A NEW CHAPTER ON THE CARE AND STERILIZATION OF SURGICAL INSTRUMENTS.

BY

**HAROLD BURROWS, M.B.(Lond.), B.S., F.R.C.S.**

### THE NURSES' PRONOUNCING DICTIONARY

2/- post free.

OF MEDICAL TERMS AND NURSING TREATMENT.

**Compiled by HONNOR MORTEN.**

NEW EDITION. Revised and Edited by MARY I. BURDETT.

Write for Catalogue of Nursing Manuals, which will be sent post free on application.

**The Scientific Press Ltd.,**

Publishers of Nursing Text-Books, Charts, and Case-Books of all Descriptions.

28 & 29 SOUTHAMPTON STREET, STRAND, LONDON, W.C.

